# Supplementary material for: Unexpected complexity of everyday manual behaviors
Source: Nat Commun. 2020 Jul 16;11:3564. doi: 10.1038/s41467-020-17404-0 (PMC7367296; doi:10.1038/s41467-020-17404-0)
Supplement: Supplementary file 1 — Supplementary Information [file 41467_2020_17404_MOESM1_ESM.pdf]

Unexpected complexity of everyday manual behaviors

Yan et al.

Supplementary Information

Supplementary Figures

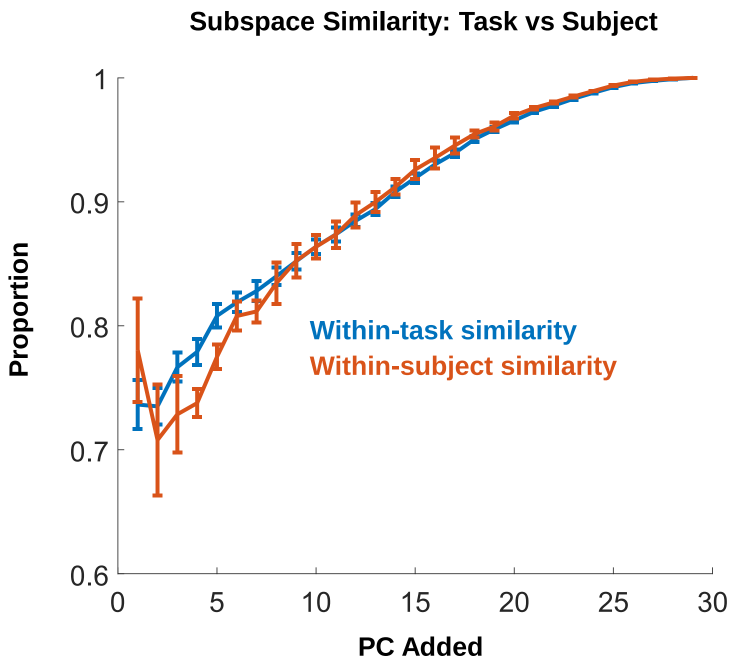

Supplementary Figure 1. Mean cross-projection similarity between kinematics subspaces (see Methods for more details) within and across tasks. Briefly, cross-projection similarity refers to how much variance from one dataset can be explained by a given number of dimensions (PCs) of the other. Within-task similarity compares the kinematics from different subjects performing the same task and within-subject similarity compares kinematics from the same subjects doing tasks (grasping and ASL). Mean of 31 within-task comparisons (28 from grasp + 3 from ASL) and 3 within-subject comparisons (from 3 subjects who did both grasp and ASL tasks); error bars denote the standard error of the mean (SEM)

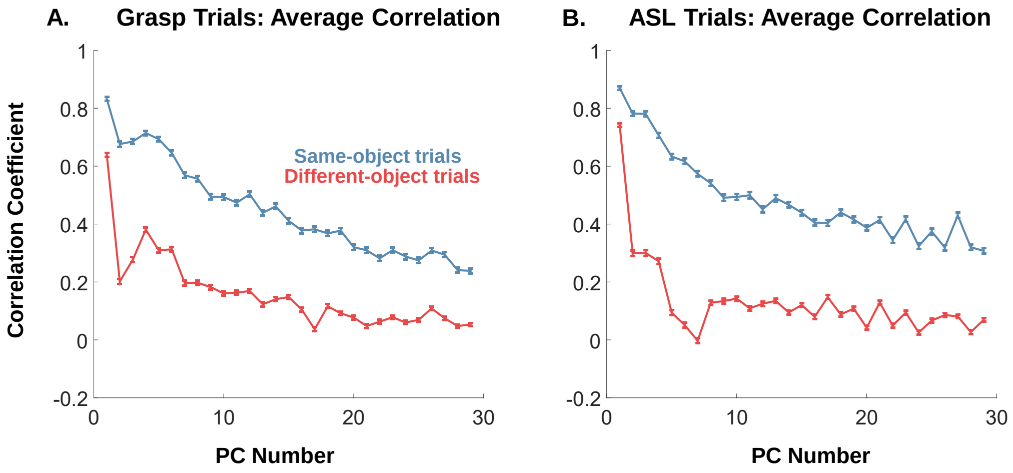

Supplementary Figure 2. Consistency of kinematics. A| Mean correlation between kinematics obtained on different grasp trials with the same object (blue) and on different grasp trials with different objects (red) projected onto the same PC, averaged across 8 subjects. The within-object correlations are systematically higher than their shuffled counterparts (Wilcoxon two-tailed signed-rank test, n = 58 (paired values at 29 dimensions), signrank = 435, p=2.56e-06). B| Same analysis for ASL trials from 3 subjects. Again, the within-object correlations are consistently higher (Wilcoxon two-tailed signed-rank test, n = 58 (paired values at 29 dimensions), signrank = 435, p=2.56e-06). Error bars denote the SEM.

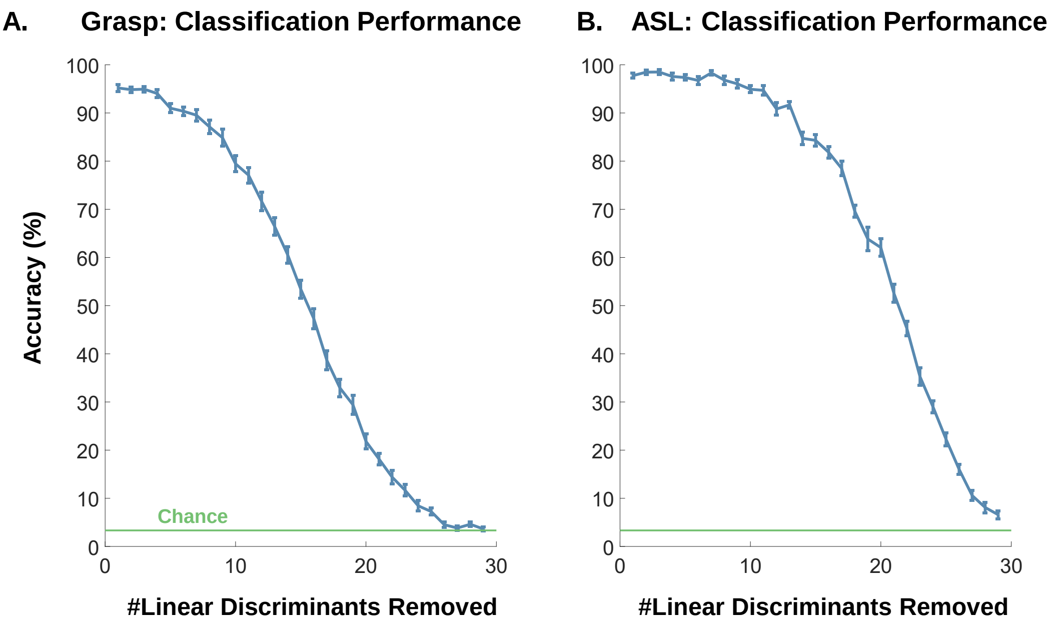

Supplementary Figure 3. Classification performance based on a progressively reduced number of linear discriminants. A| Mean classification accuracy for grasped objects as the dimensions identified by LDA are removed in decreasing order of Fisher's coefficient (ratio of between class variance to within class variance), averaged over 8 subjects. B| Mean classification accuracy of ASL postures (from 3 subjects, 5 repetitions each subject) as LDA dimensions are removed in the same way. Error bars denote the SEM.

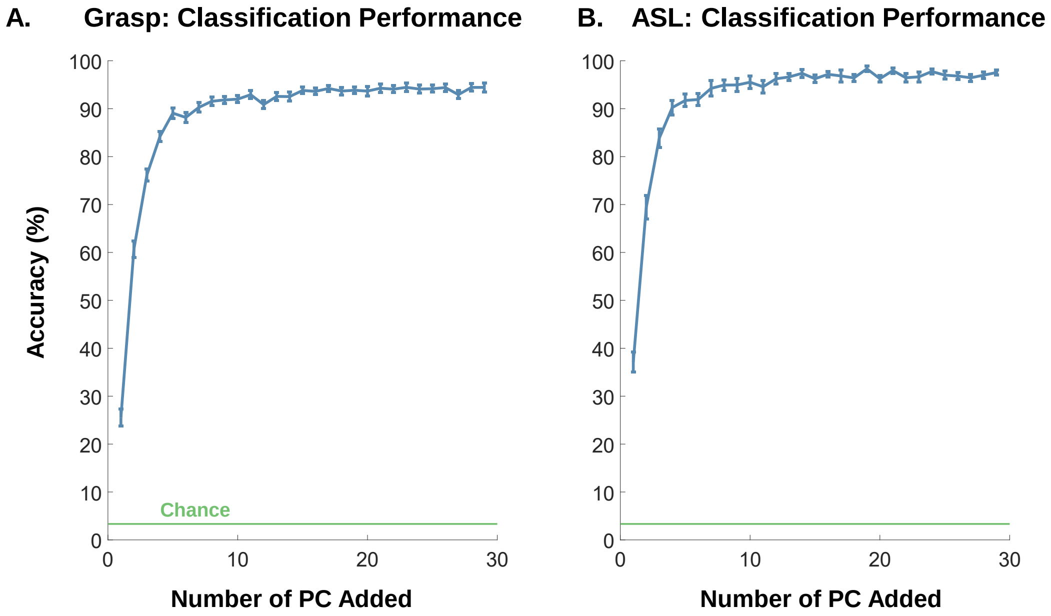

Supplementary Figure 4. Classification performance with progressively larger subspaces. A| Mean classification accuracy for grasped objects as PCs are added in descending order of variance explained (blue curve), averaged over 8 subjects. Green line denotes chance performance. B| Mean classification accuracy for ASL signs vs. number of PCs, averaged over 3 subjects. Error bars denote the SEM.

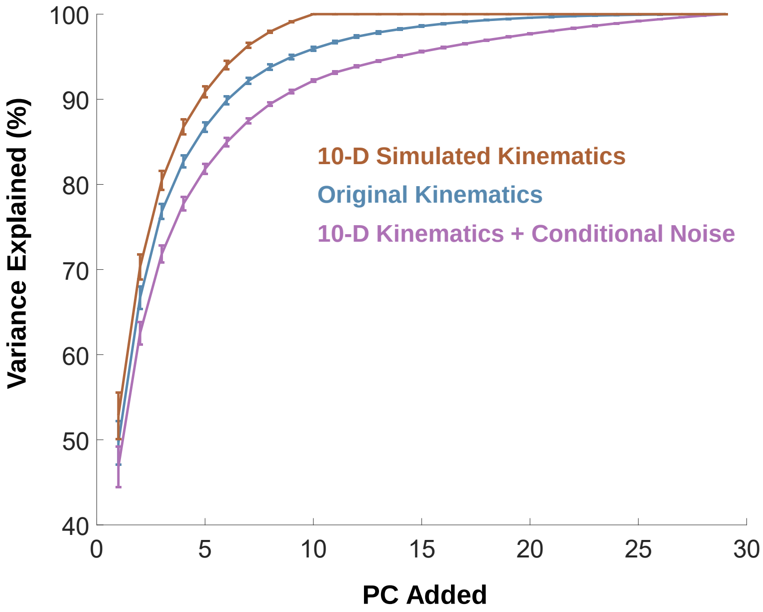

Supplementary Figure 5 Cumulative scree plot for the low dimensional kinematics with condition-specific noise. Cumulative percentage of variance explained as a function of the number of PCs for the original kinematics data, 10-D denoised kinematics, and 10-D denoised kinematics + conditional noise. Cumulative percentage is averaged over 8 subjects. Error bars denote the SEM.

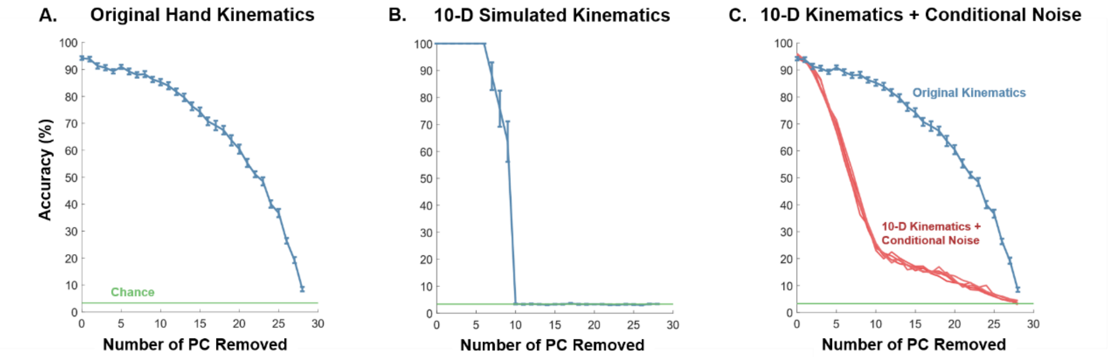

Supplementary Figure 6 Classification based on simulated low-dimensional kinematics with condition-specific noise. A| Mean classification performance of grasped objects based on the original kinematics, averaged over 8 subjects. B| Mean classification performance based on 10-dimensional de-noised kinematics. In brief, the data is denoised by taking 1 trial for each object and replicating the trial for 5 times (thereby removing all variability across trials). C| Mean classification based on the simulated (denoised, low-dimensional) kinematics to which conditional noise has been added (red curves). Each red curve represents the classification results with a conditional noise generated using a different seed (5 repetitions in total). SEMs are too small to be visible. We include the result using the original hand kinematics (blue curve, same as panel A) for comparison. Error bars denote SEM.

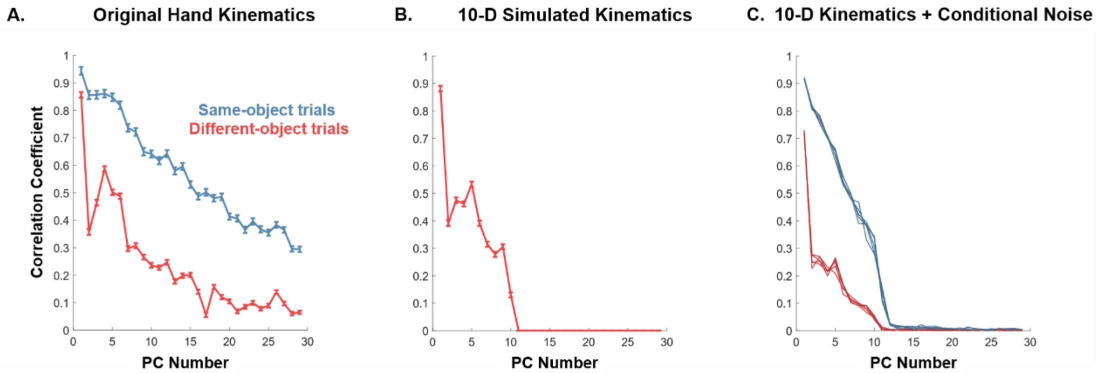

Supplementary Figure 7 Structure in the simulated kinematics. A| Mean correlation between measured kinematics on pairs of same-object grasp trials and different-object grasp trials projected onto individual PCs, averaged over 8 subjects (same as Supplementary Figure 2A). B| Mean correlation coefficients for denoised kinematics reconstructed using only 10 PCs. Here, the same-object correlations are not shown because all same-objects trials are identical given our denoising procedures. Beyond the 10th dimension, the 10-D simulated kinematics have no variance and so we set the correlations to 0. C| Mean correlation coefficients for the simulated low-dimensional kinematics with added condition-specific noise. Blue curves: same object trials. Red curves: different object trials. Each red/blue curve represents the mean correlation coefficient (from the grasp trials of 8 subjects) with conditional noise simulation generated using a different seed (5 repetitions in total). Error bars denote SEM.
