# Supplementary material for: Unexpected complexity of everyday manual behaviors
Source: Nat Commun. 2020 Jul 16;11:3564. doi: 10.1038/s41467-020-17404-0 (PMC7367296; doi:10.1038/s41467-020-17404-0)
Supplement: Supplementary file 3 — Reporting Summary [file 41467_2020_17404_MOESM3_ESM.pdf]

nature research

Corresponding author(s): Sliman Bensmaia
Last updated by author(s): May 26, 2020

Reporting Summary

Nature Research wishes to improve the reproducibility of the work that we publish. This form provides structure for consistency and transparency in reporting. For further information on Nature Research policies, see Authors & Referees and the Editorial Policy Checklist.

Statistics

For all statistical analyses, confirm that the following items are present in the figure legend, table legend, main text, or Methods section.

n/a	Confirmed	
☐	☒	The exact sample size (n) for each experimental group/condition, given as a discrete number and unit of measurement
☐	☒	A statement on whether measurements were taken from distinct samples or whether the same sample was measured repeatedly
☐	☒	The statistical test(s) used AND whether they are one- or two-sided<br>*Only common tests should be described solely by name; describe more complex techniques in the Methods section.*
☐	☒	A description of all covariates tested
☐	☒	A description of any assumptions or corrections, such as tests of normality and adjustment for multiple comparisons
☐	☒	A full description of the statistical parameters including central tendency (e.g. means) or other basic estimates (e.g. regression coefficient) AND variation (e.g. standard deviation) or associated estimates of uncertainty (e.g. confidence intervals)
☒	☐	For null hypothesis testing, the test statistic (e.g. F, t, r) with confidence intervals, effect sizes, degrees of freedom and P value noted<br>*Give P values as exact values whenever suitable.*
☒	☐	For Bayesian analysis, information on the choice of priors and Markov chain Monte Carlo settings
☒	☐	For hierarchical and complex designs, identification of the appropriate level for tests and full reporting of outcomes
☒	☐	Estimates of effect sizes (e.g. Cohen's d, Pearson's r), indicating how they were calculated

Our web collection on statistics for biologists contains articles on many of the points above.

Software and code

Policy information about availability of computer code

Data collection	We used Vicon Nexus 2.2.3 to collect all kinematics data.
Data analysis	We used Vicon Nexus 2.2.3 (process raw camera data), OpenSim 3.3 (inverse kinematics) and MATLAB 2017a (Mathematical Analysis). We only developed custom MATLAB code (Vicon Nexus is a commercial software and openSim is open-source) and they are available on Github (https://github.com/yyan-neuro/BensmaiaLab/tree/master/HandKinematics).

For manuscripts utilizing custom algorithms or software that are central to the research but not yet described in published literature, software must be made available to editors/reviewers. We strongly encourage code deposition in a community repository (e.g. GitHub). See the Nature Research guidelines for submitting code & software for further information.

Data

Policy information about availability of data

All manuscripts must include a data availability statement. This statement should provide the following information, where applicable:

- Accession codes, unique identifiers, or web links for publicly available datasets
- A list of figures that have associated raw data
- A description of any restrictions on data availability

The hand kinematics data recorded in this study has been deposited in the Bensmaia lab repository on Github (https://github.com/yyan-neuro/BensmaiaLab/tree/master/HandKinematics). We also provide the data underlying each figure as a Source Data file (SourceData.rar). The data underlying Figures 3A, 5A-B, 6A-B and Supplementary Figures 1, 2A-B, 3A-B, 4A-B, 5, and 6A-C are provided in "Source Data.xlsx". The kinematics trace data underlying Figures 2, 3B and 4A-D are provided as a MATLAB file "ASL and Grasp.mat". The data underlying Supplementary Figure 7A-C are provided as "Supplementary Figure 7.mat".

Field-specific reporting

Please select the one below that is the best fit for your research. If you are not sure, read the appropriate sections before making your selection.

☒ Life sciences ☐ Behavioural & social sciences ☐ Ecological, evolutionary & environmental sciences

For a reference copy of the document with all sections, see nature.com/documents/nr-reporting-summary-flat.pdf

Life sciences study design

All studies must disclose on these points even when the disclosure is negative.

Sample size	We did not pre-determine sample sizes. We collected hand kinematics data from every human participant who signed up for the study (8 in total) and had each of them performing at least 30 distinct hand tasks. This generated a sufficiently large dataset (360 distinct hand motions) in line with the common practice in the literature.
Data exclusions	We did not exclude data from any of the subjects.
Replication	For each grasped object / ASL signs, we ask the subjects to repeat the hand motions for 5 times. In the classification analysis, we used a trial-level leave-one-out classification paradigm, so each classification is repeated 5 times with random allocation of training and testing data. All attempts at replication were successful.
Randomization	Randomization is not applicable in our data collection. We recorded the hand movements of one subject in each session and each subject had only one session. We used random sampling with repetitions in our classifcation analysis.
Blinding	Blinding is not applicable to our study. We only instruct the subjects to perform familiar hand movements (e.g. grasp a certain object) at their own discretions. We did not provide any information that may alter the subjects' natural hand movements.

Reporting for specific materials, systems and methods

We require information from authors about some types of materials, experimental systems and methods used in many studies. Here, indicate whether each material, system or method listed is relevant to your study. If you are not sure if a list item applies to your research, read the appropriate section before selecting a response.

Materials & experimental systems

n/a	Involved in the study	
☒	☐	Antibodies
☒	☐	Eukaryotic cell lines
☒	☐	Palaeontology
☒	☐	Animals and other organisms
☐	☒	Human research participants
☒	☐	Clinical data

Methods

n/a	Involved in the study	
☒	☐	ChIP-seq
☒	☐	Flow cytometry
☒	☐	MRI-based neuroimaging

Human research participants

Policy information about studies involving human research participants

Population characteristics	The study invovles 8 human subjects , 1 female and 7 males, ranging from 21 to 40 years old. All subjects were in good health and were not undergoing treatments that might affect their hand movements. All subjects are right handed.
Recruitment	Subjects were recruited through online and offline advertisements. All subjects are students at the University of Chicago. The recruiting scheme did not introduce any bias in their hand movements.
Ethics oversight	The study is approved by the Institutional Review Board of the University of Chicago.

Note that full information on the approval of the study protocol must also be provided in the manuscript.
